# IFP35 Is a Relevant Factor in Innate Immunity, Multiple Sclerosis, and Other Chronic Inflammatory Diseases: A Review

**DOI:** 10.3390/biology10121325

**Published:** 2021-12-14

**Authors:** Roberto De Masi, Stefania Orlando, Francesco Bagordo, Tiziana Grassi

**Affiliations:** 1 Complex Operative Unit of Neurology, “F. Ferrari” Hospital, 73042 Casarano–Lecce, Italy; dmsrrt@gmail.com; 2 Laboratory of Neuroproteomics, Multiple Sclerosis Centre, “F. Ferrari” Hospital, 73042 Casarano–Lecce, Italy; 3 Laboratory of Hygiene, Department of Biological and Environmental Sciences and Technologies, University of the Salento, 73100 Lecce, Italy; francesco.bagordo@unisalento.it (F.B.); tiziana.grassi@unisalento.it (T.G.)

**Keywords:** multiple sclerosis, IFP35, IFN-induced genes, zinc-finger proteins, bZIP, leucine-zipper proteins

## Abstract

**Simple Summary:**

In this review, we focused on the emerging role of IFP35, a highly conserved leucine zipper protein from fish to humans, with a still unknown biological function. The considered literature indicates this protein as a key-pleiotropic factor reflecting JAK-STAT and DAMPs pathways activation in innate immunity-dependent inflammation, as well as in the physiology and general pathology of a wide range of phylogenetically distant organisms. These findings also indicate IFP35 as a biologically relevant molecule in human demyelinating diseases of the central nervous system, including Multiple Sclerosis, and other organ-specific chronic inflammatory disorders.

**Abstract:**

Discovered in 1993 by Bange et al., the 35-kDa interferon-induced protein (IFP35) is a highly conserved cytosolic interferon-induced leucine zipper protein with a 17q12-21 coding gene and unknown function. Belonging to interferon stimulated genes (ISG), the IFP35 reflects the type I interferon (IFN) activity induced through the JAK-STAT phosphorylation, and it can homodimerize with N-myc-interactor (NMI) and basic leucine zipper transcription factor (BATF), resulting in nuclear translocation and a functional expression. Casein kinase 2-interacting protein-1 (CKIP-1), retinoic acid-inducible gene I (RIG-I), and laboratory of genetics and physiology 2 Epinephelus coioides (EcLGP2) are thought to regulate IFP35, via the innate immunity pathway. Several in vitro and in vivo studies on fish and mammals have confirmed the IFP35 as an ISG factor with antiviral and antiproliferative functions. However, in a mice model of sepsis, IFP35 was found working as a damage associated molecular pattern (DAMP) molecule, which enhances inflammation by acting in the innate immune-mediated way. In human pathology, the IFP35 expression level predicts disease outcome and response to therapy in Multiple Sclerosis (MS), reflecting IFN activity. Specifically, IFP35 was upregulated in Lupus Nephritis (LN), Rheumatoid Arthritis (RA), and untreated MS. However, it normalized in the MS patients undergoing therapy. The considered data indicate IFP35 as a pleiotropic factor, suggesting it as biologically relevant in the innate immunity, general pathology, and human demyelinating diseases of the central nervous system.

## 1. Introduction

IFP35, also called IFI35, is an interferon-induced leucine zipper protein discovered in 1993 by Franz-Christoph Bange et al. in a cDNA library of monolayer culture of interferon (IFN) stimulated HeLa cells [1]. In 1995, Melissa A. Brown et al. first localized its gene in the 17q12-21 chromosomal region, during the characterization of the physical map of a 500-kb region of genomic DNA, containing the BRCA1 gene [2]. 

Although the regulatory activity of zipper proteins was already known, the function and its intimate mechanism of action had not been characterized by authors. Since then, little has been added to that knowledge, and the function of IFP35 still remains largely unknown. Although the stimulating properties of IFNs were already known in other chemical species, not all the functions of IFP35 were thought to depend on IFNs, and the independent ones have only been characterized recently. In fact, IFP35 was neglected for a long time, but recent studies have clarified its involvement as an intracellular signal transducer among the IFN-related signaling pathway, but above all, as an extracellular proinflammatory factor, driving the innate immunity activation. Even more recently, several works have demonstrated the IFP35 involvement in general pathology, not only concerning virus-induced infection or inflammatory experimental conditions of sepsis, but also in the chronic inflammatory human diseases of the skin, joints, kidneys, and central nervous system (CNS). The quality and the increasing number of these observations suggest that IFP35 is an important trigger factor in promoting human cell injury during the pathogen invasion, as well as the innate immunity dependent inflammation in organ-specific human diseases. In turn, innate immunity was and still is largely underestimated in literature, as compared to adaptive immunity. However, it is known that the definitions of *adaptive* and *innate* are merely scholastic and refer to functional compartments, in order that there is a well-defined link between them, in molecular and cellular terms.

Cellular terms mainly consist of monocytes for the reticulo-endothelial system, dendritic cells (DC) for the integumentary system, and microglial cells for the CNS. These elements serve as professional antigen presenting cells (APC), as well as T cells, which are immersed in an inflammatory *milieu*. In addition, they are responsive to several foreign antigens through the plasmalemmal or cytoplasmic toll-like receptors (TLR), resulting in the production of inflammatory cytokines, costimulatory molecules CD80, CD86, and type I interferon by the interferon regulatory factor (IRF) activation. Due to their bridge position between the innate and adaptive immune response, monocytes play a fundamental role in the physiopathology of the tissue damage and in inflammatory diseases, also considering that the adaptive response follows and cannot take place without the innate one. Furthermore, the inflammatory dependent upregulation of the co-stimulatory factors on autoreactive T cells escaping intrathymic negative selection is thought to be related to the loss of peripheral immune tolerance, a biological *primum movens* to different target organs for chronic inflammatory diseases. 

Regarding IFP35, these premises are even more important, given the following considerations regarding monocytes: These elements have plasmalemmal IFN receptors and provide a prompt response to them. In addition, they can actively release IFP35 to the extracellular space during cellular injury and pathogen invasion. Finally, different types of damage associate molecular pattern (DAMP) molecules have been found to activate TLR-mediated inflammatory response and recently, IFP35 has also been proven to be an in vivo proinflammatory DAMP [3]. Moreover, IFP35 appears to have a more relevant involvement in Multiple Sclerosis (MS), due to the chronic inflammatory nature of the disease, and to the IFN-beta-1a usage as the first line disease modifying treatment (DMT) of this condition. Consequently, IFP35 has been recognized recently as a biomolecular marker of disease activity and treatment response in MS [4]. 

Considered together, these data show IFP35 as a more important trigger factor in the biology and physiopathology of inflammation than has been represented until now in the literature, and worthy of further investigation. The general update of our knowledge regarding this molecule is the aim of the present work, as well as the recent evidence of IFP35 involvement in the human pathology. 

## 2. The bZIP Overview

Eukaryotic genes are differentially regulated by a complex group of DNA-binding proteins, depending on the environmental and developmental signals. Transcription factors (TFs), polymerases, and nucleases belong to this group, as well as histones. Therefore, the existence in nature of highly conserved TF homeodomains belonging to different ranks of phylogenetic development, but afferent to the same cellular functions is not surprising. This is the case with basic leucine zipper (bZIP) proteins. These are ubiquitous proteins with a DNA-binding function conferred by their basic amino acidic and dimeric ZIP sequences folding, precisely similar to the zip in the presence of the alpha helix.

At present, eight major families of mammalian bZIPs have been described, according to their ligands, including the C/EBP, TRE, CRE, CRE-like, PAR sites, and the so-called small musculoaponeurotic fibrosarcoma (Mafs) protein. The latter comprehends three members called MafG, MafF, and MafK, with as a common structure, beyond the bZIP, an extended homology region that enables them to make a broader contact with the DNA, resulting in obligatory heterodimeric partners for cap ‘n’ collar and Bach transcription factors, that cannot bind to DNA as monomers [5].

Homeodomains of leucine zipper proteins have been found in a large variety of eukaryotic organisms, ranging from yeast to humans, and more than 80 of these protein families have already been identified as belonging to the four DNA-binding families, among these, about 750 are zinc-finger proteins, including the leucine-zipper proteins, such as IFP35. 

All of the known tridimensional TF motifs of DNA-binding domains are, in turn, classifiable according to their structure: Helix-turn-helix, helix-loop-helix, zinc fingers, beta-scaffold, and the bZIP. The latter which characterizes leucine-zipper proteins, is first described by Landschulz et al. in 1988 [6]. The amino acid segment that promotes dimerization is the basic-region (BR) leucine zipper motif of the homologous class of main eukaryotic transcription factors, although also described in the prokaryotic. 

In summary, this domain consists of an uninterrupted alpha-helix with a BR binding the DNA, and a C-terminal leucine zipper (LZ) motif responsible for the dimerization [7,8]. The amphipathic quality conferred by ZIP motifs rules the specific interaction with the DNA, but also with other proteins, through the modulation of the Van der Vaals weak interactions. In particular, bZIP coiled coils contain 3- and 4-residue repeats, resulting in an amphipathic pattern of alpha-helices, in which amino acids are designated from the ‘*a*’ to ‘*g*’ positions and constitute heptad repeats of tertiary conformation. Positions ‘*a*’ and ‘*d*’ are generally hydrophobic, forming a zigzag pattern of knobs and holes interlocking with a similar pattern on another strand to perform the hydrophobic core of the quaternary structure, while charged residues in positions ‘*e*’ and ‘*g*’ contribute to electrostatic interactions of the external microenvironment [9]. 

In the case of leucine zippers, leucines are predominant at the ‘*d*’ position of the heptad repeat of the tertiary structure. These residues pack against each other every second turn of alpha-helices, inducing a hydrophobic region of residues in the ‘*a*’ position between two helices. This conformation defines the bZIP domain as a universal structure for the protein-protein interaction. This bZIP structure was defined through several methods, including fluorescence, NMR-, CD- and Raman-spectroscopy, calorimetric or hydrodynamic methods, and small-angle X-ray scattering, as well as other biophysical techniques [10]. However, it refers to the dimeric or heterodimeric state of the protein. In fact, these proteins can assume a different conformational state in the presence or absence of DNA, with a dynamic conformational entropy state, ranging between the structured shape in its presence and the disordered, non-random one in its absence. Therefore, the BR is intrinsically unstructured/disordered in the absence of DNA, but the presence of the alpha helix induces its folding in a Y-like tertiary rearrangement [11]. 

This DNA interaction system exists in a highly conserved manner in a variety of eukaryotes organisms. Based on the nucleotide position +2, different kinds of ACGT- containing elements are classified as A-box, C-box, G-box or T-box. This variability and affinity of binding is enhanced by the presence of disordered/unstructured regions between the BR and heptad repeats, resulting in structural plasticity that allows proteins to adopt more than one conformation, depending upon the ligand. 

A bZIP database (bZIPDB) containing worldwide information on protein interactions and TF-target gene relationships of more than 49 human bZIP TFs has been developing for some years now [12]. In addition, the presence of ions between the dimer and the DNA [7], the redox status [13], and the DNA flexibility [14] also affect the DNA-binding. This lack of definite conformation enhances the regulatory possibilities by better exposing lateral chains and facilitating post-translational modifications [15]. This is the mechanism thought to expand locations on the genome to which bZIP transcription factors can bind and regulate gene expression. 

Finally, it is known that the bZIPs functions linked to the DNA-binding activity depend on their translocation to the nucleus from the cytoplasm. In fact, bZIPs can be transferred to the nucleus only after a proteolytic cleavage of the N-terminal trans-membrane domain. Then, they need a nuclear localization signal (NLS) to target the nucleus, consisting of two clusters of lysines/arginines located within the BR [16]. In addition, phosphorylation can control the bZIP cytoplasmic retention [17] and its nuclear import [18]. Coherently, bZIP17 [19], bZIP28 [20], and bZIP60 [21] have been found to be membrane associated in Arabidopsis.

Regarding the regulation of the 56 bZIP genes known in humans, the upregulation or mutation of leucine zipper regulatory proteins, such as *c-fos* and *c-jun,* as well as *myc*, *max,* and *mxd1* may cause cancer. The cAMP response element-binding protein (CREB) involved in the neuronal plasticity is thought to induce neurodegeneration in humans, if downregulated. Furthermore, the binding activity of bZIP53 to the 2S2 albumin promoter is significantly improved when combined with bZIP25 or bZIP10 [22]. Conversely, the DNA-binding activity of the bZIP1 is prevented in combination with bZIP63 or bZIP10 [23]. This is a stoichiometric adjustment, but a fine regulation in the transactivation capacity of bZIP gene expression is attributed to other enhancing elements, including the clock protein CCA1 [24] and the inhibiting ones, such as BBX25 [25]. The ABI5 homodimers activate gene expression, whereas the EEL homodimer and ABI5-EEL heterodimer repress it [26], while types S and C bZIPs coordinate the activation of metabolic response to low-energy stress in combination with SnRK1 [27,28,29,30]. 

Finally, another mechanism of bZIP protein regulation is the phosphorylation. It can affect dimerization, and the specific DNA-binding of bZIPs sequences can be prevented by the addition of a phosphate group into the BR. This induces a negative charge, resulting in repulsive forces with the DNA [31,32]. This is the case of ABF3, whose phosphorylation creates a binding site for a 14-3-3 protein, which protects the same ABF3 from rapid turnover [33]. Likewise, the phosphorylation of HY5 prevents its interaction with E3-ubiquitin-protein ligase COP1 and the consequent degradation [34]. Signal transducer and activator of transcription (STAT) proteins must be phosphorylated to bind DNA, as well. Through these control systems, the cells can adapt to the different chemical environments and their changes. For examples, Zn deficiency increases the transcription of bZIP23 and bZIP19 [35]; osmotic stress induces bZIP53 and bZIP10 [36]; drought and salt in Arabidopsis and tomatoes upregulate the ABA-responsive element binding protein (AREB) subfamily of bZIPs; and sucrose represses the translation of the S1 subgroup of bZIPs (bZIP1, bZIP2, bZIP11, bZIP44, bZIP53) [37,38]. The abundance of some bZIPs, including GBF1 [39], ABF1, and ABF3 [40] or TGAs [41] even can express the specific control of the protein turnover after the protein synthesis. 

## 3. IFP35

IFP35 is an IFN-induced 35-kDa leucin-zipper protein with cytoplasmic localization and nuclear interaction, but unknown function. However, fractionation studies and immunofluorescence microscopy have failed to detect any association of IFP35 with specific organelles [42]. This molecule was discovered as a differentially expressed protein (DEP) among the IFN-treated MS in comparison to the untreated and healthy subjects [43,44]. In addition, more recently, it has been included in the DAMPs mediated by TLR, that trigger the innate immunity and inflammatory response [3]. Moreover, other observations have found this molecule to be a critical player in inflammation, as well as in general and human pathology. Consistent with its taxonomy, IFP35 is a highly conserved regulative protein, acting in different ranks of the evolutionary scale, among more than 900 IFN-stimulated genes. The study of this protein improves the current knowledge regarding the antiviral response and the innate immunity-dependent inflammation in animals and humans. 

However, due to the inaccessibility to the blood brain barrier (BBB) and the constitutive low neuronal responsiveness to IFN, the CNS responses triggered by this molecule are very low as proven in vivo by the lack in expression of stimulated IFP35 and Apolipoprotein L9 displaying antiviral activity [45]. This concept also applies to the therapeutic type I IFNs that express a merely peripheral mechanism of action (MoA). Therefore, there are important functional differences in the IFN response mounted by CNS and the other non-nervous tissues that must be considered.

### 3.1. The Structure of IFP35

Even though the notions regarding the tertiary structure are still incomplete, they are more relevant than the ones regarding the function of IFP35. No specific studies have been conducted concerning the latter, nor have small angle scattering or NMR techniques been applied. In addition, what we know comes from the study of the primary structure by Franz-Christoph Bange et al. [1], as well as those by N. Deannie Lee and Jun Chen et al., who described in subsequent studies the domains of the protein [46,47]. The amino acidic sequence analysis revealed a 288-amino acid protein with four N-myc-interactor (NMI)/IFP35 domains (NIDs) (among these, only two have been studied) and a leucine zipper motif in proximity to the NH-terminus (aa 5–25). This motif is constituted by a heptad repeat of five leucines in a predictable period and alternating with two leucines in *e* position, resulting in a structured alpha helix with a 4,3 hydrophobic repeat, which realizes coiled-coil sequences to form a continuous hydrophobic interface. Four out of eight amino acids in positions *b* and *d* are charged, thus theoretically allowing an inter-helical interaction [48]. This bZIP motif mediates protein-protein interaction and dimerization, allowing for the positioning and orientation of DNA-binding domains on palindromic sites. However, the latter function is only putative, given the absence of the DNA-binding basic domain at the extreme amino terminus. This is a peculiar structural condition among leucine zipper proteins, making IFP35 an exception element in the belonging family and it is thought to confer a negative regulatory function on the transcriptional process. 

**Figure 1:**
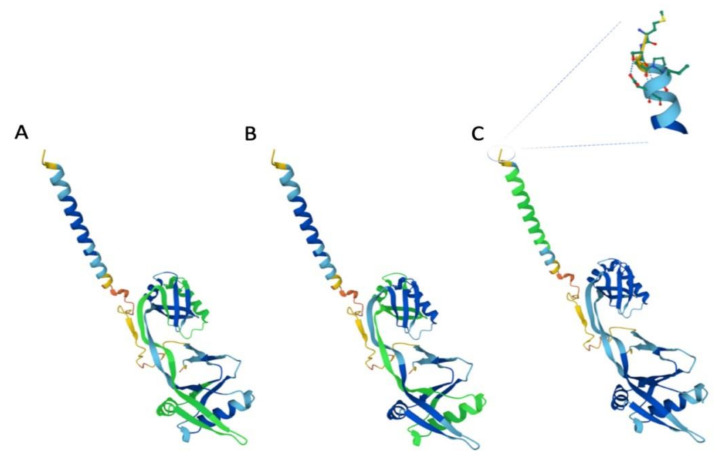
The tertiary structure of IFP35. Note, in green, respectively NID1 (**A**), NID2 (**B**), and leucine zipper motif (**C**). In C, also the N-terminus with its amino acid residues is represented in an exposed manner. (Figure reproduced from https://www.uniprot.org/uniprot/P80217/protvista, accessed on 7 September 2021).

More recently, the Western blotting analysis has demonstrated that IFP35 functionally interacts with NMI through its NID1 (aa 81–170) and NID2 (aa 183–266) (Figure 1).

These domains show 46% homology and 25% identity to two homologous NID domains of NMI (NID1, aa 103–192; NID 2, aa 201–292), which mediate and constitute the high molecular mass complex between IFP35 and NMI. In fact, the knockdown of NMI significantly mitigated the inhibitory effect of IFP35 [46,47]. The remaining regions of the protein have not been studied. 

### 3.2. The Function of IFP35

**Figure 2:**
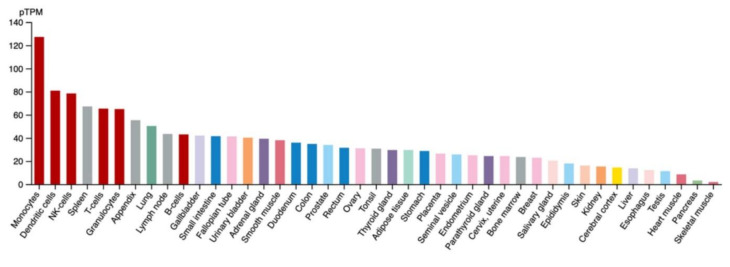
The human protein atlas. Note the preponderant expression of IFP35 in the tissue and cells of the immune system. The *pTPM* protein-transcripts per million. (Figure reproduced from https://www.proteinatlas.org/ENSG00000068079-IFI35/tissue, accessed on 7 September 2021).

The biological function of IFP35 remains largely unknown. However, it can be assumed that the protein is a negative regulator of transcription considering its peculiar structure and analogy to other similar peptides with the helix-loop-helix (HLH) motif, but lacking a functional basic region. The first notion regarding the involvement of this molecule in the innate immunity is suggested by its preponderant expression in the spleen and immune cells in the humans (Figure 2), as well as in fish gills, working as a mammalian mucosal-associated lymphoid organ (MALT)-like structure.

However, more concrete and reproducible data come from the original article [1], in which IFP35 is shown to be an IFN-inducible factor with a related cytoplasm-nucleus translocation in HeLa cells fractioned lysates and to regulate antiviral gene transcription and cytokine response. This interferon-inducible property of IFP35 has also been confirmed in vivo using therapeutic IFN-beta-1a, IFN-beta-1b, as well as IFN-gamma in mice, resulting in final NF-kB inhibition [3]. Interferon molecules work as antiviral glycoproteins via the JAK-STAT cascade after the plasmalemmal receptor activation and the immune response is highlighted by the significant increase of the expression level of interferon related signaling molecules, including interferon regulatory factor 3 (IRF3), IRF7, IRF5, interferon-stimulated gene 15 (ISG15), and IFP35 precisely. IFN-gamma induces IRF-1 expression via STAT1, and the enhanced IRF-1 expression further leads to an increase of IFP35 expression. This molecular pattern, so called the IFN interactome, is demonstrated to come from fish and shellfish and from mammalians and humans, as well. However, the main mammalian network is thought to be composed of IRF1 and STAT1. 

In addition, although IRF-3 is constitutively expressed in all cell types, IRF-5 and IRF-7 are predominantly expressed in cells of lymphoid origin and can be further induced by type I IFN. Specifically, after the link between the IFN molecule and the specific receptor (IFNGR), the ligand-dependent dimerization of these subunits rapidly activates the associated JAK by auto phosphorylation, which provide docking sites for STAT proteins, such as, type I IFN-induced tyrosine kinase 2 (TK2) and JAK1 phosphorylate C-terminal tyrosine residues of Y701 in STAT1 and Y690 in STAT2. At this point, a supramolecular complex constituted by STAT1, STAT2, and IRF9, named IFN-stimulated gene factor 3 (ISGF3), undergoes nuclear translocation and binds IFN-stimulated response elements (ISREs), resulting in more than 900 interferon stimulated genes (ISGs) transcription. The ISGs core gene includes 95 genes shared among lymphocytes B, T, and monocytes. Among these, we find the IFP35 and NMI as immune response elements [3]. It is not surprising that in 2000, Jun Chen et al. found the highly conserved NMI as the major cytoplasmic heterodimeric partner of IFP35, forming a 200–300 kDa high molecular mass complex [47,49]. The NMI protein, in fact, interacts with many TFs containing HLH and bZIP domains, but also with all STATs chains (except for STAT2), resulting in the upregulation of their transcripts in response to IL-2 and IFN-gamma. The NMI/IFP35 dimerization prevents the proteasome-dependent degradation of IFP35 [47] and is crucial for the amplification of IFNs’ physiologic effects, nuclear translocation, and consequent functional expression of this molecule. However, the IFP35/NMI heterodimerization occurs reversibly in a phosphorylation-dependent manner, in order that de-phosphorylation on seven IFP35 Ser/Thr potential sites correlates with the IFN-induced 100–200 kDa complex formation [49]. Although NMI was also shown to interact with c-Myc and N-Myc in mammalian cells, the role of this modulation has not yet been documented, but the Myc function in cell growth, transformation, and apoptosis is well known. In fact, NMI was found to inhibit the proliferation and metastasis of cancer cells [50]. By contrast, it had also reported that NMI promotes hepatocellular carcinoma progression via BDKRB2 and MAPK/ERK pathway [51].

Finally, the upregulation of NMI during cell injury and pathogen invasion suggests that its role in tissue inflammation and coherently NMI^−/−^ mice in LPS-induced sepsis models were partially protected [3]. In 1996, X. Wang et al. discovered the basic leucine zipper transcription factor (BATF) belonging to the AP-1/ATF superfamily, as the first interactor of IFP35 [52]. We do not know the intimate biological significance of this interaction, but BATF can dimerize with the oncogene Jun. In addition, the BATF/JUN transcriptional activity induces pro-inflammatory IL4 expression, as well as Th17 differentiation, resulting in a critical pathogenic player in many disease models, also including the CNS ones. It was demonstrated that the transgenic BATF^−/−^ mice immunized with myelin oligodendrocyte glycoprotein peptide 35–55 (MOG35-55) express complete resistance to experimental autoimmune encephalomyelitis (EAE) [53], and the BATF/JUN complex is proposed as an anti-inflammatory therapeutic target [54]. Zhang et al., in 2007, and more recently Lin Fu et al., in 2019, demonstrated that the casein kinase 2-interacting protein-1 (CKIP-1) could bind the IFP35 that competes with NMI for association, during monocyte immunity. This interaction system plays an important role in acute and subacute inflammation control via the IFN-gamma and IL-2 [55,56]. The CKIP-1, in fact, is a multiple biologic interactor containing an N-terminus pleckstrin homology (PH) domain and a C-terminus LZ motif with dimerization capacity. Therefore, the ratio of NMI to CKIP-1 ultimately controls the IFP35 levels, which is itself a short-lived protein, avoiding its proteasome-induced degradation. This suggests that the CKIP-1 is an involved factor in cytokine signaling and serves as a physiological regulator of IFP35 and NMI in vivo.

Another IFP35 regulatory protein acting with a similar modality is the E3 ubiquitin ligase tripartite motif protein 21 (TRIM21). This protein conjugates the ubiquitin on target proteins and has been shown to be an important regulator of antiviral signaling [57]. In fact, A. Das et al., in 2015, found that TRIM21 interacts with coiled coil domains of IFP35 and NMI conjugating the K63-linked polyubiquitin chains exclusively on the latter [58]. This ubiquitination occurs via the SPRY domain of TRIM21 on the NMI lysine 22 residue and is required to stabilize the NMI-IFP35 complex and to promote its negative regulatory function on the innate antiviral response. Interestingly, TRIM21 has been found as an autoantigen in several autoimmune diseases, including Rheumatoid Arthritis (RA), Systemic Lupus Erythematous (SLE), and Sjogren’s syndrome. In addition, the anti-TRIM21 antibodies are recognized as diagnostic and prognostic markers of these diseases [59,60].

Furthermore, Das et al. revealed, in 2014, an interaction between the cytoplasmic RNA sensor retinoic acid-inducible gene I (RIG-I) and IFP35. This interaction resulted in negative feedback with downregulation of the same RIG-I due to K48-linked ubiquitination and degradation, through the proteasome machinery [61]. This IFP35-dependent negative regulation of RIG-I has been demonstrated in vitro using the experimental model of U373MG astrocytoma cells treated with polyinosinic-polycytidylic acid (poly I:C), a viral mimic [62], and in vivo by viral assessment studies on fish as well as humans, and on human autoimmune diseases, as described below (see the Fish section). Another IFP35 interacting factor according to the negative feedback regulation was described in 2016 by Yepin Yu et al. [63]. This is a homolog laboratory of genetics and physiology 2 (LGP2) from marine fish, orange spotted grouper Epinephelus coioides (EcLGP2), with an important member of RIG-I-like receptors (RLRs). Similar to RIG-I, EcLGP2 is a pathogen sensing element with activating properties of innate immune responses and the upregulation of the latter decreases the expression level of interferon related molecules, including IRF3, IRF7, ISG15, IFP35, MXI, MXII, and melanoma differentiation associated gene 5 (MDA5), suggesting a negative modulation of interferon immune response by EcLGP2 and a consequent negative regulation of antiviral response in vitro. Importantly, cytoplasmic TRIM21 is also known to bind Fc regions of antibodies complexed with neutralized virus particles and belongs, along with RIG-I and EcLGP2, to the innate immunity receptors, as well [64]. In 2017, Zhikai Xiahou et al. found IFP35 and NMI to be a DAMP [3]. These extracellular DAMPs are recognized by a pattern recognition receptor (PRR), including the monocytic plasmalemmal TLR4. Unlike TLR1-2-5-6 that utilizes the molecular adaptor MyD88 to activate NF-kB and AP-1, TLR3 utilizes the adaptor TRIF to activate IRF3 and IRF7. TLR4 can utilize both ways. TLR7 and TLR9 are endosomal and utilize MyD88 to activate NF-kB and IRF7. It is known that MyD88 and TRIF can both activate IRF and NF-kB. IRF activates and upregulates IFN genes resulting in the antiviral state. On the contrary, MyD88 activates and upregulates NF-kB resulting in a proinflammatory response based first on the innate immune activation, and then on the adaptive one, with cytokines secretion (TNF, IL-1, IL-6), chemokines secretion (CCL2, CXCL8, etc.), adhesion molecules secretion (E-selectins), and eventually costimulatory molecules (CD80, CD86) upregulation. Specifically, monocytes/macrophages and PRRs, as well as TLRs belonging to the innate immunity system, trigger the innate immunity-dependent inflammation by the activation of NF-kB, but also costimulatory molecules resulting in APC induction and eventually, in the adaptive immunity-dependent inflammatory effector. The latter is constituted by pro-inflammatory cytokines and cells, including IL-17, IL-21, and Th17, as well as Th1, resulting in the granulocyte-macrophage colony stimulating factor (GM-CSF) release with granulocytes recruitment and further lymphocytes differentiation on inflamed tissues [65]. Moreover, NMI and IFP35 both amplify the inflammatory response, as actively released by macrophage to extracellular space during pathogen invasion and cell injury. Therefore, DAMPs contribute to the controlling of the inflammatory responses according to a fine balance between *pathogenic noxa* elimination and the organ damage. Interestingly, another recognized DAMP is the S100 calcium-binding protein [66]. The same one serving as a *primum movens* in the epitope spreading mechanism of MS.

On the one hand, all of these notions indicate an incomplete knowledge regarding the functions of IFP35. On the other hand, they also indicate its involvement in the link between inflammation and immune responses. In fact, the presence of DAMPs is associated to cellular death, that can be apoptotic or necrotic. Apoptotic cellular death implies local inflammation, where DAMPs are largely lacking, unlike necrotic cellular death, in which DAMPs and inflammation are preeminent and interplaying elements. It is known that TLR-mediated monocytes activation, in the presence of DAMPs, can induce the inflammatory environment and expression of costimulatory molecules, resulting in APC-dependent maturation of DC, as well as monocytes and the adaptive immune response elicitation. In addition, of note, this DAMP-dependent elicitation of immune responses and inflammation is temporally and spatially spread, grading from apoptosis to necroptosis that are focal events, to pyroptosis that is an organic process and, eventually, to panoptosis that has systemic features. This inflammatory spread reflects the DAMP amount release, ranging respectively from the focal one to the systemic and uncontrolled one. This is the case of the IFP35 and NMI, as well. Furthermore, IFP35/NMI has been reported as a Caspase-insensitive complex during the apoptosis process. However, this in vitro property does not yet have a defined biological significance. Further insights on TLRs and DAMPs will exceed the aims of the present work, but they can be noted in these articles [65,67,68,69].

**Figure 3:**
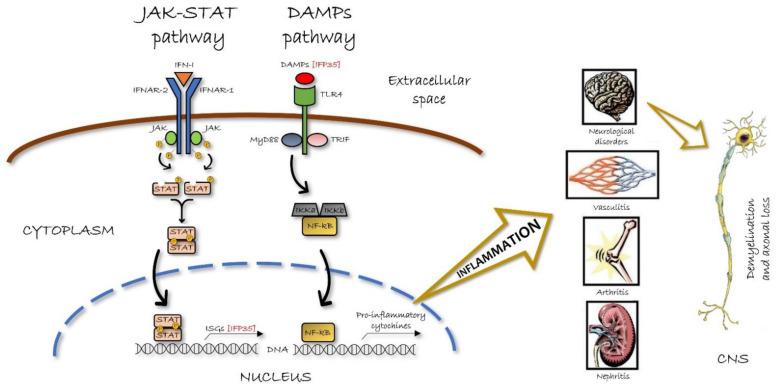
Mechanistic figure of JAK-STAT and DAMPs pathways involving IFP35. In the JAK-STAT pathway, the link between type I IFN molecule (IFN-I) and its receptor (IFNAR-1/IFNAR-2) induces the ligand-dependent dimerization of intracellular subunits, rapidly activating, in turn, the associated Janus kinase (JAK) by auto phosphorylation. Then, the activated dimer of signal transducer and activator of transcription (STAT) undergoes nuclear translocation, resulting in different interferon stimulated genes (ISGs) transcription, such as 35-kDa interferon-induced protein (IFP35). In the DAMPs pathway, the extracellular IFP35 serves as a damage associate molecular pattern (DAMP) molecule, recognized by Toll like receptor 4 (TLR4). The latter utilizes both molecular adaptors MyD88 and TIR domain-containing adaptor protein inducing interferon beta (TRIF) to activate nuclear factor kappa B (NF-kB) or interferon regulatory factor (IRF), respectively. While the IRF activation upregulates IFN genes resulting in the antiviral state, the MyD88 activation leads to an interaction between NF-kB and IkB kinases (IKK complex). Then, the activated NF-kB is translocated into the nucleus where it acts as a transcription factor of various pro-inflammatory cytokine genes. This results in a pro-inflammatory response based first on the innate immune activation, and then on the adaptive one. The immune response elicitation finally increases the inflammatory demyelination and axonal loss in the central nervous system (CNS) and tissue damage in the other organ-specific chronic inflammatory diseases.

All of these data converge towards the dichotomous concept regarding the IFP35 function. On the one hand, this molecule serves as a DAMP and expresses its proinflammatory activity via TLRs and the adaptor TRIF, eventually resulting in NF-kB enhancement. On the other hand, IFP35 serves as an IFN-induced factor via the JAK-STAT cascade and IRFs eventually resulting in NF-kB inhibition, with the net effect depending largely on the type of *pathogenic noxa* and its transduction factors. The human protein atlas in Figure 2 shows the main expression of IFP35 in monocytes, dendritic cells, NK-cells, lymphocytes T, and spleen. This is the lecture key for the interpretation of physiological and pathological findings regarding IFP35 and its biology. Figure 3 shows the representative mechanism of action of both JAK-STAT and DAMPs pathways involving IFP35.

## 4. IFP35 in The Pathology 

Since the dawn of time, humans and animals have evolved in an environment characterized by pathogen and host-pathogen interaction. This co-evolution selected PRRs as sensing molecules for the presence of pathogens, resulting in the activation of innate immune responses. PRRs were subdivided into four major families, including TLRs, NOD-like receptors (NLRs), C-type lectin receptors (CLRs), and RLRs [70]. The latter family was found to be composed of RIG-I, MDA5, and the laboratory of genetics and physiology 2 (LGP2). IFP35 is known to interact with TLR4 and LGP2. Furthermore, increasing evidence indicates IFPs as emerging molecules involved in pathological and physiological immune response. This is especially true for IFP35.

### 4.1. IFP35 in The Virus-Induced Pathologies

#### 4.1.1. Bovines

In 2008, J. Tan et al. demonstrated the antiviral role of IFP35 in bovine foamy DNA virus (BFV) infection [71] for the first time. These results indicate that IFP35 may represent a novel pathway in the antiviral action of interferon. Foamy viruses can infect mammals (such as cattle, monkeys, cats, horses, and humans) and belong to the Spumaretrovirinae subfamily of the Retroviridae. They infect hosts by encoding two functional promoters, the long terminal repeat (LTR) and the internal promoter. These two elements are regulated through a key transactivator bovine protein (BTas), a DNA binding protein that transactivates both proteins. The authors, demonstrated by the yeast two-hybrid screening and the co-immunoprecipitation study, a physical interaction between the IFP35 and BTas resulting in a molecular complex undergoing a nuclear translocation. In this case, the BTas molecule works as a chaperon for the nuclear entry and the cytoplasm-to-nucleus translocation of IFP35, very similar to that induced by the interferon treatment. This IFP35/BTas interaction reduces the BTas-dependent activation of the BFV LTR in a dose-dependent way, eventually inhibiting the viral replication. This evidence confirms the idea that the N-terminal leucine zipper motif of IFP35 confers a negative regulation of transcription, as suggested by the absence of the DNA-binding basic domain at the extreme amino terminus.

#### 4.1.2. Human Models of Influenza

Due to the current echo-systemic upheaval and pervasive anthropization, the cross-species viruses’ transmission is raising major public health concerns. This is now the case with the COVID-19, and it was also the case with bird and swine flu. The H3N2 subtype swine influenza virus (SIV) was used to characterize dynamic cellular responses to infection in human inoculated A549 cells. The two-dimensional electrophoresis (2DE) study coupled with the Western blot analysis of nuclear and cytoplasmic fractions evidenced several DEPs, including IFP35 and NMI, upregulated in the infected study pool. Confocal microscopy confirmed the cytoplasmic co-localization of these species and their nuclear translocation as effects of viral infection. Finally, the Ingenuity Pathway Analysis found DEPs as belonging mainly to the NF-kB and IFN signaling networks [72]. Serum IFP35 was also found to confer susceptibility to H5N1 and H1N1 infections in mice hosts, as a modulating agent in pathogenic innate immune response. TLR stimulation of IFP35^-/-^ knockout mice challenged with H1N1 induced a reduced cellular infiltration and decreased cytokines production, including IL-12p40, in hematopoietic cell compartment [73]. These data confirm the critical role of IFP35 in cytokine production and the inflammatory process, triggering and exacerbating during viral infection.

#### 4.1.3. Fish

IFP35 has been studied in fish purely for research and applicative purposes, in the effort to ameliorate the marine aquaculture reproduction of rockfish in the Asian-Pacific regions, including the Republic of Korea, as well as in the snakehead fish and groupers cultures in China and Singapore. In fact, viral and bacterial infections are continuously a cause of huge economic loss for these businesses. The main finding in this field is the characterization of two conserved NIDs homology domains at C-terminus in the IFP35 gene of mandarin fish Siniperca chuatsi, as well as a high percentage of disordered or intrinsically unfolded regions. Furthermore, although IFP35 is a phylogenetically highly conserved factor, it is lacking in the leucine zipper motif [74]. This conformation confers it with structural flexibility, reducing the entropic cost of protein-protein interactions resulting in the known function of IFP35. NIDs at 162e248 and 259e346 express common features shared by other IFP35 homologs, with homo- and hetero-dimerization capacity. This applies to mandarin fish, rockfish, and other available fish sequences of IFP35 in the NCBI databases, such as P0056216, KKF31655, P6060, P003442654, NP001135293, and KPP1121 [75]. Other differences in the amino acid sequence alignments of IFP35 in fish were also considered and compared to different species to obtain the phylogenetic tree of IFP35 that was distinguished in reptiles, birds, and mammalians. This suggests a low identity of the IFP35 amino acidic sequence between homologs belonging to different species. 

These studies found IFP35 as a ubiquitously expressed factor in all of the examined tissues, with the highest expression in the blood and spleen of rockfish and teleost. However, this distribution is very different in the infection spleen and kidney necrosis virus (ISKNV)-infected mandarin fish with the highest IFP35 expression in the gills, suggesting it as a MALT feature [74]. All of the transcriptional and quantitative mRNA assessments, both in vitro and in vivo, evidenced the importance of IFP35 as an interferon-induced factor in the first-line host defense system against viruses and bacteria. The authors confirmed the IFP35 cytoplasmic co-localization and heterodimerization with NMI and the nuclear translocation kinetic on Western blot and immunoprecipitation methods using poly I:C or IFNs and LPS as triggers. Unlike other cases, only the transcriptomic profiles of striped snakehead cells (SSN-1) infected with snakehead vesiculovirus (SHVV) identify IFP35 as a positive factor for SHVV replication by acting on the RIG-I like receptor-signaling pathway [76], but this is the unique case in which IFP35 negatively regulates the host antiviral response. However, the greatest knowledge was obtained from groupers. In fact, J. Liu et al. evidenced the helicase DDX41 as an intracellular DNA sensor, crucial for triggering type I interferon, resulting in the antiviral response to the Singapore grouper iridovirus (SGIV) or red spotted grouper nervous necrosis virus (RGNNV) [77]. EcDDX41 (DDX41 homolog from the orange spotted grouper Epinephelus coioides is mainly localized in the gills and significantly overexpressed during SGIV and RGNNV infections. This upregulation is associated with the nucleo-cytoplasmic shuttling of the molecule and induces the overexpression of IFP35, ISG15, ISG56, Viperin, and MXI, and suppresses the viral gene transcription, suggesting the antiviral activity of EcDDX41. This antiviral property was confirmed in other species as shrimp, ducks, and humans as well, but in the grouper it is further modulated by the Beclin-1. This autophagic regulator has recently been found to colocalize in endoplasmic reticulum and exerts antiviral activity through modulating IFN and inflammatory responses, as well as virus-induced cell death [78].

#### 4.1.4. Humans

Human hepatitis C virus (HCV) and immunodeficiency virus (HIV) are common etiological factors of chronic infection diseases in humans. During these chronic processes, it is vital to tightly balance host protective elements and viral replication mechanisms since uncontrolled innate immune activity is detrimental to the host. In all cases, IFP35 and ISGs are IFN-induced pro-host factors for the containment of viral replication. In particular, the upregulation of IFP35 and miRNA99 was found to constitute a fingerprint in predicting the sustained virological response (SVR) in HCV infection, mainly in the non-G1 viral genome type [79]. However, although IFP35 is involved in the IFN-induced antiviral functions, its overexpression suppresses the activation of IFN-beta and ISG56 promoters, suggesting a negative control feedback in non-responder patients [61]. Moreover, the upregulation of the S1PR1 induces internalization and lysosomal degradation of IFNAR1, resulting in the auto-amplification disruption of the interferon molecular pattern. 

Similarly, USP18, a ubiquitin-specific peptidase, is stimulated by JAK-STAT signaling and provides negative feedback to this pathway by binding IFNAR2, resulting in the promotion of viral replication [80]. IFP35 is differentially expressed in the HIV infection, as well. In particular, logistic regression models correlated the upregulation of interferon response monocytic genes, including IFP35, IFNAR1, and STAT1 to depression, in smokers. These results suggest that smoking augments the inflammatory effects of the HIV infection on the immune response in monocytes, also inducing clinical depression [81].

The role of IFP35 has also been studied in the acute viral infections. Regarding the vesicular stomatitis virus (VSV), the knockdown of IFP35 expression by siRNA abolished pEGFP-N1-2C and pEGFP-N1-Nmi-induced activation of type I interferon promoters [82]. This enhanced the VSV replication, suggesting the critical role of IFP35 in the type I interferon response. Once again, the authors demonstrated the cytoplasmic IFP35 interaction with NMI using the confocal microscopy and the co-immune precipitation. However, the direct cellular infection with VSV induces the upregulation of ISGs, including LGP2 and IFP35 that negatively regulate the antiviral response. This negative regulation is mediated by RIG-I, which is a well-known PRR for negative-strand viruses, such as VSV and the mandarin fish virus. Specifically, IFP35 negatively regulates the RIG-I-mediated antiviral pathway by two modes: (i) By phosphorylating its CARD domains by phosphatases PP1-alpha/gamma and (ii) by a proteasome-dependent degradation via K48-linked ubiquitination [83]. In this case, IFP35 acts as an inhibitor of the RIG-I antiviral signaling pathway, highlighting the little-known negative regulation of cellular antiviral responses. This active downregulation of type I IFN via the RIG-I was described in mandarin fish and stomatitis viral pathologies, but also has a physiological relevance in human pathology, particularly in SLE and psoriasis, which, indeed, have been found to be associated with a negative regulation of IFN [84]. 

### 4.2. IFP35 in The Organ-Specific Chronic Inflammatory Diseases

There is a physiopathological link between chronic viral infections and autoimmunity. This link is mediated by the inflammatory environment that causes cross-reactivity, resulting in the innate-immunity activation and self-antigens exposure to the professional APC, according to the molecular mimicry mechanism and consequent elicitation of the immune response. This cross-reactivity is described in Chikungunya, Epstein-Barr, and Zika virus infections associated with acute arthritis of unknown origin. However, a viral hypothesis was formulated for many other organ-specific chronic inflammatory diseases, including MS, RA, and SLE. 

#### 4.2.1. Rheumatoid Arthritis

RA is a chronic, inflammatory, autoimmune disease characterized by the presence of inflammatory immune cells in the diarthrodial joints, resulting in synovial hyperplasia with final bone structure loss and functional impairment. The pathogenesis of RA is described as depending on the netosis phenomenon of neutrophils, owing to citrullinated and carbamylated antigens exposure with the production of autoantibodies and immune complex formation, acting as endogenous activators of IFN-alpha production and ISGs [85]. Pathogenic antibody formation and immune complexes attract the CD4^+^ Th2 lymphocytes from the blood stream, which are, in turn, linked to autoantibody generation, to response polarization through the interaction of CD40 and CD40L, as well as CTLA4 and, also, proinflammatory cytokines liberation, such as type I and II IFNs, TNF-alpha, and IL-6. Van der Pouw first described the elevation of IFN levels as pathogenically related to this autoimmune disease versus controls [86]. Since then, other studies have confirmed the upregulation of type I interferon and regulated genes by this molecule in the pathogenesis of the disease. IFP35 and MXA were overexpressed in peripheral blood mononuclear cells (PBMCs) of RA diseased patients, and statistically correlated with the anti-carbamylated proteins antibodies (Anti-CarP) and anti-citrullinated peptide antibodies (ACPA) titers, suggesting a pathogenic involvement of ISGs and confirming the antibodies’ pathogenic role [87]. Furthermore, the over expression of IFP35 and IFP44 was found in pregnant woman affected by RA [88]. These molecules were highly expressed in the second trimester and postpartum, compared to the non-pregnant and non-diseased pregnant women, but without correlation to the disease activity. This lack of correlation has been attributed to an IFP35 expression due to the endogenous triggers, such as ones deriving from tissue changes during placentation and delivery, not to the joint inflammation resurgence or a pathogen invasion. 

#### 4.2.2. Endothelial Inflammation 

The endothelium is not considered to be a bystander element, in both acute and chronic inflammatory pathologies. These imply the alteration of the surface molecule repertoire of endothelial cells, adhesion and transmigration of mononuclear cells, secretion of cytokines by invading cells during emperipolesis, and secondary changes in gene expression by smooth muscle and endothelium. Many studies have addressed this topic in the literature, but few of these include IFP35. Using gene expression microarrays, IFP35 has been found to be involved in the endothelial response to TNF-alpha- and IL-1-beta-induced inflammation in experimental conditions [89]. In addition to the chemical species, such as TLR4, as well as Class I and II of major histocompatibility complex (MHC), known to be associated with anti-viral or anti-bacterial activity and complement components, a novel response network involving the signal transduction and transcription regulation category was described, which is IFP35, precisely. However, although at the moment this is context-specific data, which is not clinically transferable, the importance of IFP35 has been confirmed recently by Dongdong Jian et al. [90]. The authors used a C57BL/6 mice model of carotid artery injury, with findings in IFP35 adenovirus-transduced mice, the re-endothelization rates at days 3 and 7 were significantly lower compared to that in null adenovirus-transduced mice, specifically 5% and 35% vs. 20% and 50%, respectively.

Moreover, in the mice model of permanent middle cerebral artery occlusion (PMCAO), IFP35 was found to reduce the p56 protein in the nucleus, while affecting the production of p-p65 in the cytoplasm [91]. At the same time, the signaling pathway of NF-kB was reduced, as well as VEGF, CD105, and bFGF, resulting in the inhibition of angiogenesis and endothelial cells replication in mice with acute cerebral infarction. This has also confirmed the NMI role in complexing with IFP35. In fact, the knockdown of NMI significantly mitigated the inhibitory effect of IFP35 on endothelial cells proliferation and migration. The authors commented that these findings indicate that IFP35 is a promising target for the prevention and treatment of post-injury vascular intima hyperplasia.

#### 4.2.3. Drug-Associated Profiles of IFP35

Few studies assessed transcriptomic modifications in organisms undergoing drugs. The need to ameliorate the farming fish and cattles enhanced our knowledge in this field. However, the human pathology was considered, as well. Ractopamine hydrochloride (RHC) is supplemented to cattles to increase carcass weight for commercial purposes. As a beta-adrenergic stimulator, RHC was thought to be associated with the stress response, resulting in metabolic inflammatory pathways activation. Actually, the blood transcriptomic analysis after 35 days of supplementation evidenced the IFP35, as well as TYROBP and TP53INP1 as DEPs without a systemic dysregulation of inflammatory pathways, given no induction of TNF-alpha, IL-6, and IL-10 [92]. Therefore, the RHC-induced upregulation of IFP35 is associated with the cattle weight increase, but not to the systemic inflammation. On the basis of known immunomodulatory effects of vitamin D, the 1.25(OH)2D3-enriched diet of yellow catfish was assessed in vivo and in vitro concerning the resistance of fish after an immune challenge with LPS and poly I:C. The real-time quantitative PCR demonstrated an anti-inflammatory modulation of VD3/VDR-type I IFN axis genes, including VDR, IRF3, IFN-a, JAK1, STAT1, IFI56, and IFP35. Specifically, IFP56 and IFP35 were significantly upregulated in both the kidney and spleen in response to the dietary vitamin D3 contents, as well as increased serum lysozyme, catalase, and SOD activities, resulting in a final enhanced innate immunity response against pathogens [93]. 

Regarding humans, the upregulation of IFP35 assumes the opposite biologic significance, according to the anti-inflammatory reaction in Statin administration and the pro-inflammatory one in the Imiquimod application. Statins’ pleiotropic effects are known, including the anti-inflammatory, antioxidant, antithrombogenic, and neuroprotective ones. These properties have been also confirmed in subjects with chronic obstructive pulmonary disease (COPD) who take Statins. Differential gene expression analysis also revealed, in addition to LDLR and SC4MOL genes pertaining to the lipid homeostasis, the trafficking-regulating CXCR2 as a downregulated gene in treated COPD patients, as expected [94]. On the other hand, the IFP35 overexpression detected by real-time PCR during Imiquimod topically applied to skin lesions caused by the basal cell carcinoma (sBCC), Bowen’s disease or cutaneous T cell lymphoma, was associated with many other proinflammatory elements with clinical improvement. These elements include the so-called IFN-alpha signature (IFP35, OAS1, ISG20, MxA, and IRF7), chemokines and chemokine receptors (IL-1-beta, IL7R, etc.), killer cell-associated receptors (NK-p46, KIR3DL2, etc.) and others [95]. In effect, Imiquimod directly activates innate immune effectors through TLR7/MyD88-dependent pathways and induces costimulatory molecules (CD80 and CD86) of the dendritic cells, resulting in the release of Th-1 immunity specific cytokines, such as IFN-alpha, TNF-alpha, and IL-12 [96]. This innate immunity-mediated topic inflammation, finally, induces an antitumor immune response. 

In short, all of these studies describe IFP35 as a signaling factor, acting in metabolic and cytokines modulation, and not only as involved in innate immune-dependent response elicitation and inflammation.

#### 4.2.4. Lupus Nephritis

SLE and its most common and severe complication, lupus nephritis (LN), have been studied recently through an advanced microarray data meta-analysis, using a large scale pathway-based approach. This approach provides a useful technique for grouping and combining disparate microarray datasets to identify a strong (meta) signature specific for pathology. The metasignature of SLE includes several differential genes involved in the IFN, TLR signaling pathways, and inflammatory cytokines. In addition, a significant hypomethylation was found on the CpG sites of genes EIF2AK2 (cg14126601 and cg17326313) and IRAK3 (cg19995654, cg12866960, and cg07914866). These are coding genes for TLR, and recently they have been found hypomethylated, as well as IRF, in SLE patients with LN [97]. The IFP35 was overexpressed and its gene hypomethylated, together with those of ISGs and others, including CD80, OAS1, IFI44, STAT1/2, JUN, CCL2, MX1, IL1R2, and CXCL1. Specifically, IFP35 was suggested as having a pathogenic role in the innate immunity-dependent glomerular inflammation, triggered by TLR3-induced type II IFN activation, resulting in modulation of RIG-I/CCL5 and MDA5/CXCL10 [98]. This cascade is triggered by a viral or “pseudoviral” antigen, serving as a molecular trigger and pathogenic *primum movens* with an unknown identity and provenience, as generally postulated for autoimmune diseases. IFP35 has an important role, suggesting its putative therapeutic target also in this case.

#### 4.2.5. Skin Diseases 

Although few studies deal with the issue, we mention them since they describe different patterns of IFP35 in two opposite conditions: The apoptotic one in atopic dermatitis (AD) [99] and the proliferative one in Sezary syndrome (SS) [100]. Common inflammatory diseases of the skin, from eczema to AD and chronically relapsing skin disease, recognize the enhanced apoptosis of keratinocytes as the main pathological feature [101]. The mRNA array analysis from AD patients revealed increased IFN-gamma-induced apoptosis of keratinocytes as the key factor. Consequently, the over expression of IFP35 and other ISGs was observed, including CCL5, CCL8, IFITM1/2, as well as apoptosis-related genes NOD2, DUSP1, and ADM. The pattern of IFP35 is antiproliferative and proapoptotic in this case.

Unlike AD, keratinocytes in psoriasis undergo hyperproliferation, but we do not have any IFP35 reports on this pathology. In SS, a lymphoproliferative condition of the skin, so-called fungoid mycosis, was found, resulting in a leukemic form of cutaneous T cell lymphoma. The SS transcriptome analysis from PBMCs revealed the promyelocytic leukemia zinc finger protein (ZBTB16) as the most upregulated gene and IFP35, as well as IRF3, as key factors significantly downregulated, resulting in a proliferative effect. Obviously, these data need to be expanded, but they clearly suggest the IFP35 involvement also in apoptosis and cell proliferation control.

#### 4.2.6. Mice Models of Sepsis and Inflammation

Many notions concerning inflammation, as well as sepsis and its modulation by IFP35 have derived from the studies applied to models of LPS-induced sepsis in mice. In particular, in vivo LPS-mediated triggering at different dose regimens in a TLR-dependent manner is known to activate the innate immunity and consequent inflammation, mimicking bacterial invasion. On the other hand, poly I:C-mediated triggering can mimic viral invasion. In both models, molecular adaptors MyD88 and TRIF involve the IRF and NF-kB cascade with final co-activation, balancing between type I IFN expression genes such as IFP35 and pro-inflammatory elements, including TNF-alpha, IL-1, CCL2, CXCL8, CD80, CD86, E-selectine, etc. [102]. Moreover, the correlating effect is well known between LPS, as well as IFP35 and the mice survival curve, as expected. Interestingly, IFP35 has also been demonstrated to be a DAMP molecule [3]. Soluble extracellular IFP35 and NMI can directly stimulate peripheral monocytes, acting on their plasmalemmal TLR4, activating proinflammatory cytokines and NF-kB, resulting in enhanced inflammation and even release of IFP35 and NMI, as well. Coherently, NMI^-/-^ and IFP35^-/-^ knockout attenuate the inflammatory response, improving the survival rate of mice in LPS-induced sepsis models.

Consistent results were obtained by the murine model study of appendicitis and appendectomy (AA) which, when carried out at younger ages, protects against colitis development later [103]. In the AA model, the expression of IFNs and IFN-related genes in the distal colon was initially highly expressed and subsequently decreased over time, from 3 to 28 days post-AA. In particular, the IFP35 expression decreased from 1.43- to 1.03-fold over this time range, reflecting the reduced inflammatory involvement of the colon. In a murine total-body irradiation model, the jejunum IFP35 expression decreased in G-003M (a combination compound of Podophyllotoxin and Rutin against radiation injuries) pre-treated mice in comparison to the untreated ones, indicating the reduced inflammation after radiation exposure using the 2DE [104]. In this model, the upregulation of thioredoxin domain-containing protein 17 (Txndc17) contributed to inflammatory mitigation, through the negative modulation of TNF and NF-kB. 

Finally, we cite the case of Diamond Blackfan Anemia (DBA). DBA is a rare genetically inducted human anemia, which represents a model of an in vivo inflammation, and thus a proper *experimentum naturae*. In fact, haploinsufficiency of ribosomal proteins due to mutations in ribosomal genes induces bone marrow inflammatory microenvironment changes, leading to red cell aplasia in diseased subjects [105]. This inflammatory microenvironment is thought to induce IFN-gamma and ISGs, including IFP35, as well as NMI, GBP1, and PSMB8/9/10 that are overexpressed. These data confirm, once again, the involvement of IFP35 in inflammation and cell replication.

#### 4.2.7. Multiple Sclerosis

MS is the most common chronic juvenile demyelinating disease of CNS with an inflammatory and degenerative component, and it is also the main cause of non-traumatic disability in the young. Although many DMTs have been available in the last decade, the first approved and still used treatment remains the type I IFN-beta-1a/b. Roberto De Masi et al., in 2009, first described 2DE coupled to the MALDI-TOF analysis, IFP35 from PBMCs as a DEP in relapsing remitting MS patients (RRMS) [43]. They found this protein highly expressed in naive MS patients compared to healthy controls. Moreover, IFP35 was about 5-fold overexpressed in IFN-treated RRMS patients in comparison to the untreated ones. A statistical analysis also highlighted a direct correlation between the IFP35 expression values and disease duration, as well as neurological disability expressed in terms of the expanded disability status scale (EDSS) score. 

In 2020, the same author described for the first time IFP35 as a biomolecular marker of disease activity and treatment response in MS [4]. Specifically, the Western blot analysis confirmed the previous proteomic results and highlighted as a non-responder to IFN therapy the RRMS patients expressing IFP35 of less than the cut-off value of 65%. Interestingly, the authors found IFP35 overexpressed also in naive patients and patients affected by a clinically isolated syndrome (the first demyelinating event of the CNS), with a significant reduction after the start of several treatments, including Dimethyl Fumarate and Natalizumab. On the one hand, all of these data suggest the property of IFP35 in reflecting the biological IFN activity. On the other hand, the data suggest the involvement in MS of the innate immunity and its DMT-mediated anti-inflammatory modulation. 

More recently, X. Jing et al. confirmed with the clinical transcriptomic that the expression of IFP35 and MNI was highly expressed in subjects affected by MS. Their in vitro experimental observations also highlighted that these factors are involved in the NF-kB-dependent activation of microglia via the TLR4 pathway and they can activate dendritic cells, resulting in naive T cell differentiation in Th1 and Th17 cells. Coherently, NMI^−/−^, IFP35^−/−^ or the administration of neutralizing antibodies against IFP35 alleviated the immune cells’ infiltration in the CNS, thus reducing the severity of EAE [106]. Together, these findings identify IFP35 and NMI as emerging targets for diagnostic and prognostic biomarkers of neuroinflammation in MS. 

## 5. Conclusions

IFP35 is a highly conserved leucine zipper protein with still unknown biological functions. Previous data have described it as a molecule reflecting the IFN activity with antiviral and antiproliferative final effects. However, convincing works have described IFP35 as a DAMP element, acting in the induction and amplification of the innate immunity-dependent inflammation. It consists of disordered domains, in addition to the HLH one, resulting in improved protein-protein interaction properties. However, the lack of DNA-binding basic domain at the extreme amino terminus of the protein confers a negative regulatory function on the transcriptional process. IFP35 heterodimerizes and co-localizes in the cytoplasm, mainly with NMI and BATF, as well as CKIP-1. These factors stabilize IFP35, thus avoiding its proteasomal-dependent degradation, also allowing for its functional expression which occurs after the nuclear translocation and subsequent activation. This proteasomal degradation, in turn, is thought to be mediated by an unknown signal.

The DNA-sensing cytoplasmic factors, RIG-I and EcLGP2 can also interact with IFP35 during the innate immunity response, expressing, in the end, negative feedback on the ISG expression and the antiviral activity. In fact, IFP35 reflects the stimulation of type I IFN via the JAK-STAT cascade, resulting in an antiviral and antiproliferative effect in the case of endogenous secretion, and an anti-inflammatory one in the case of exogenous administration, as a DMT in MS. However, IFP35 also serves as an innate immune-dependent pro-inflammatory DAMP. This finding is derived from in vivo studies on a mice model of sepsis, and it has also been confirmed in human pathology. IFP35 was highly expressed in diseased subjects suffering from LN, AR, and untreated RRMS, while it normalized in the latter group of patients undergoing therapy. Moreover, reflecting IFN activity, the IFP35 expression level predicts the disease activity and the response to IFN therapy in MS. 

This review includes, to the best of our knowledge, all of articles currently concerning IFP35. The considered data describe a molecule with pleiotropic functions, a long-acting phylogenetic key-role in innate immunity, as well as in the physiology and general pathology of a wide range of organisms, from fish to humans. Increasing evidence has rapidly shifted the role of IFP35 from being “a new leucine zipper protein” to being an innate immunity derived biomolecular marker in MS. This trend suggests IFP35 as a clinically relevant factor in human pathology, as well as in understanding the natural mechanism of innate immunity and correlated inflammation.

## Data Availability

Not applicable.

## Abbreviations

2DE	two-dimensional electrophoresis
AA	appendicitis and appendectomy
AD	atopic dermatitis
APC	antigen presenting cells
BAT	basic leucine zipper transcription factor
BFV	bovine foamy DNA virus
BR	basic region
BTas	transactivator bovine protein
bZIP	basic leucine zipper
CKIP-1	casein kinase 2-interacting protein-1
CNS	central nervous system
COPD	chronic obstructive pulmonary disease
CREB	cAMP response element-binding protein
DAMP	damage associate molecular pattern
DBA	Diamond Blackfan Anemia
DC	dendritic cells
DEP	differentially expressed protein
DMT	disease-modifying treatment
EAE	experimental autoimmune encephalomyelitis
EcLGP2	laboratory of genetics and physiology 2 Epinephelus coioides
HLH	helix-loop-helix
IFN	interferon
IRF	interferon regulatory factor
ISG	interferon-stimulated gene
LTR	long terminal repeat
LZ	leucine zipper
Mafs	musculoaponeurotic fibrosarcoma protein
MDA5	melanoma differentiation associated gene 5
MHC	major histocompatibility complex
MS	Multiple Sclerosis
NIDs	NMI/IFP35 domains
NLS	nuclear localization signal
NMI	N-myc-interactor
PBMCs	peripheral blood mononuclear cells
Poly I:C	polyinosinic-polycytidylic acid
PRR	pattern recognition receptor
RA	Rheumatoid Arthritis
RGNNV	red spotted grouper nervous necrosis virus
RHC	Ractopamine hydrochloride
RIG-I	retinoic acid-inducible gene I
RLR	RIG-I-like receptors
RRMS	relapsing remitting MS patients
SGIV	Singapore grouper iridovirus
SHVV	snakehead vesiculovirus
SLE	Systemic Lupus Erythematous
SS	Sezary syndrome
STAT	signal transducer and activator of transcription
TFs	transcription factors
TLR	toll like receptors
TRIM21	tripartite motif protein 21
VSV	vesicular stomatitis virus
